# Exploring the Path of Innovative Development of Traditional Culture under Big Data

**DOI:** 10.1155/2022/7715851

**Published:** 2022-08-29

**Authors:** Zhixian Li

**Affiliations:** Institute for Advanced Study, Confucius Academy, Guiyang, Guizhou 550025, China

## Abstract

Chinese traditional culture is the treasure of our cultural field. In the new era, it is of great significance to give traditional culture a new life and vitality. The term “big data” is hotly debated all over the world, while the development of big data is gradually occupying all aspects of the society that people are compatible with society. It is an imperative initiative to build a cultural data system by making use of big data technology, and cultural big data can make Chinese traditional culture release more vitality. This paper analyzes the new characteristics of traditional culture development from big data in helping traditional culture inheritance and innovation and proposes new ideas and creates more possibilities for the development of traditional culture. Combining with big data technology, this paper proposes an improvement to the data sparsity problem and cold-start problem of collaborative filtering recommendation algorithm and also improves the recommendation algorithm based on association rules. The association rule technique is used to compensate for the cold-start and data sparsity problems of new users often encountered by collaborative filtering techniques; the aim is to obtain recommendation results with high user satisfaction. Experiments on traditional cultural resource datasets show that the method in this paper effectively solves the data sparsity and cold-start problems that exist in traditional collaborative filtering techniques, and the recommendation accuracy surpasses that of other methods.

## 1. Introduction

Chinese excellent traditional culture has a long history and rich content and is the cultural treasure house of the Chinese nation with great profundity. Chinese excellent traditional culture has great guiding significance for the direction, path, and specific contents of socialist cultural construction with Chinese characteristics in the new era. In the postepidemic era, the situation at home and abroad is changing rapidly, and we are facing a major change in the world's development that has never been seen before [1]. Promoting the inheritance and development of excellent Chinese traditional culture and combining excellent traditional culture with the great practice of socialism with Chinese characteristics is an important path to achieve the grand goal of cultural power at present [2].

As one of the world's four ancient civilizations, China has a long history of 5,000 years and contains a profound, long-standing, and excellent traditional culture. Generations of Chinese people have used their rich experience and wisdom to nurture great cultural wealth, making it an important cultural symbol of our country, which is also the most solid foundation of the Chinese nation's heritage. What is traditional Chinese culture? Integrating the many different views and opinions in domestic academic circles, we can roughly understand it from this perspective: Chinese excellent traditional culture is the spiritual wealth that has been formed, accumulated, and preserved by the Chinese nation for thousands of years under the conditions of specific geographical location, economic base, and political system [3]. Chinese excellent traditional culture exists and continues both in the physical form of objects such as cultural relics and monuments and in the abstract form of subjects such as values, ways of thinking, ethics and morals, aesthetic orientation, and traditional customs [4]. These excellent traditional cultures conform to the objective laws of human social development, have withstood the test of time for a long time, and still have a strong influence on the development of contemporary China. Entering a new era, new conditions have emerged in the inheritance and development of Chinese excellent traditional culture, and we are faced with new challenges in the inheritance and development of Chinese excellent traditional culture.

People lack a sense of identity with the excellent Chinese traditional culture. On the one hand, they do not have a strong will to accept the excellent Chinese traditional culture themselves, and they lack a heartfelt interest in promoting the excellent Chinese culture, and they are not willing to experience it firsthand; on the other hand, because the fields carrying the excellent Chinese culture were once too formalized, they think that visiting the fields carrying the excellent Chinese culture is completing the task, rather than truly accepting and learning from the heart the cultural concepts conveyed by the fields carrying the excellent Chinese. On the other hand, because the venues carrying excellent Chinese traditional culture were once too formalized, they thought that visiting the venues carrying excellent Chinese traditional culture was completing their tasks, rather than truly accepting and learning the cultural concepts conveyed by the venues carrying excellent Chinese traditional culture from their hearts [5]. In their view, the visit to the site carrying excellent Chinese traditional culture is a set of formalized procedures of “taking pictures,” “listening to reports,” and “writing experiences,” and it invariably also invariably increases the amount of their tasks and takes up their time. The protection system of Chinese traditional culture is weak both domestically and abroad, and the state has formed legislation for cultural heritage, but the effect of supervision based on administrative laws and regulations such as the intangible cultural heritage law of the People's Republic of China is not obvious [6]. However, for a period of time, the education of excellent Chinese culture has been in a state of neglect, whether it is the calligraphy class in school education, the exchange and transmission of excellent Chinese traditional culture in family education, or the subtle indoctrination influence of excellent Chinese traditional culture itself. There are problems, and this lag has only emerged in recent years with the improvement of the understanding of excellent Chinese traditional culture [7].

The long river of the years is vast, the wheel of history rolls forward, and our country continues to prosper, all without the support of the excellent Chinese traditional culture [8]. Although our country has had its share of difficulties, we have been able to survive and revitalize our nation. The development of today's China is proving to the world that our motherland is strong; the strength of today's China also comes from the spiritual backbone of the excellent Chinese traditional culture.

The stable development of society is also inseparable from the escort of the excellent Chinese traditional culture, which has invariably formed a moral constraint for people in the society [9]. The traditional virtue of the Chinese people for thousands of years, “respecting the old and loving the young,” has invariably become a kind of moral constraint, and in public places, people always unconsciously give way to and take care of the elderly and children, which cannot be completely restrained by cold standards like laws. Once this moral constraint is formed, it will be deeply imprinted in the heart and naturally become a kind of convention, which will continue to accumulate force for the stable development of society.

In the era of big data, people have replaced paper media with the Internet and information technology, etc., which have developed into more efficient and higher-quality ways of transmitting information, and at the same time have provided greater possibilities for updating and changing the ways of transmitting traditional culture [10]. At the same time, we need to recognize that the rise of the big data era has created both opportunities and challenges for the development of traditional culture. The significant characteristics of “large capacity, multiple types, fast access, and low value density” of big data make the dissemination of good traditional culture encounter the huge impact of the current emerging culture such as network culture and fast-food culture [11, 12]. Only by making real and scientific information run faster than rumors and pseudoscience can rumors and pseudoscience lose their “market” for survival.

The main contributions of this article are as follows: first, cultural communication is an important way for innovation and development. In the era of big data, being able to recommend traditional culture-related news and knowledge based on users' hobbies or needs is a key technology. The commonly used collaborative filtering recommendation algorithm has problems such as sparse data and cold start. In this paper, we follow the idea of hybrid recommendation algorithm and incorporate the association rule-based recommendation algorithm and content-based recommendation algorithm based on collaborative filtering algorithm to improve the problems in their algorithms.

## 2. Related Works

### 2.1. The Current State of Development of Chinese Traditional Cultural Innovation

Chinese traditional culture is the “root” and “soul” of the Chinese nation, and the development of Chinese civilization for more than 5,000 years has been accompanied by specific sociohistorical conditions. As time passes, the development of traditional Chinese culture into modern society, the original cultural connotation needs to be expanded, improved, and changed, and the form needs to be innovated. Therefore, it is necessary to give the excellent Chinese traditional culture a new connotation of the times, adapt to the needs of people's lifestyle today, realize its modern transformation, and provide a recipe for further solving contemporary problems.

Culture comes from the continuous transmission and creation of generations, as reflected in a rich array of cultural and artistic works and achievements in the field of ideology, which is undoubtedly the result of long years of human practice [13]. Through long practice, many cultural features, cultural products and cultural spirits are preserved, forming specific records that continue from generation to generation, from which writing emerges. This is the practicability that is reflected in traditional culture. The practice of ancestors for generations was written, recorded, and preserved, which became the summary of practical experience and the culture that was inherited and continued, which is at the same time a process of practice rising to theory. With practice, theory can be formed; with theory, further practice can be guided [14]. The unity of theory and practice is not only the characteristic of traditional culture but also one of the most basic principles of Marxist theory. Excellent Chinese traditional culture is an important content of socialist culture with Chinese characteristics and is of great significance to economic and social development. However, in the process of promoting traditional culture, many difficulties will be found.

With the rapid development of globalization and the close cultural exchanges between countries, students are disturbed by diverse cultures from all over the world, and with the immature ability to judge and choose values, they are prone to distortion of values and loss of self. Practitioners of traditional culture have to integrate traditional culture into their daily lives. First, they should pay attention to the communication and interaction with young people, observe the feedback of young people in their lives, find out the problems, and do a good job of “guiding the way,” so as to help them form the correct value judgment and the ability to choose values. Second, practitioners of traditional culture should be good at discovering young people's interests, guiding them to carry out research in the areas of traditional culture they are interested in, and asking them to write corresponding research reports with their own feelings, experiences, and relevant knowledge. Again, it is necessary to pay attention to the timing of the introduction, which should be combined with the content of the course involving traditional culture, so as to enhance the scientific and effective education of traditional culture with the help of the systematic and logical knowledge of the course content, improve the understanding of young people about traditional culture, and strengthen the memory of the core concepts. Finally, it is important to note that a guided approach can not only be achieved by traditional culture practitioners alone but also requires young people to actively cooperate with traditional culture practitioners in their lives, to think about issues on their own, and to actively give feedback on their feelings and suggestions. In addition, young people need to continue to consolidate their knowledge, apply it in practice, explore new areas, and improve themselves. Defining something, interpreting its concept, is the basis and prerequisite for doing what follows. Culture is no different. Over the years, there have been many interpretations of the concept of culture in academic circles. However, as far as contemporary society is concerned, the fact is that few people can clearly indicate what culture actually consists of, and most people do not have a certain grasp of the connotation and extension of traditional Chinese culture, and the controversy over the concept of culture may remain only in the academic community and is not popularized [15]. The public talks about culture in general terms, what is culture, what is the excellent Chinese traditional culture, and how people should treat traditional culture, without a correct and systematic concept, not to mention the innovative development of culture.

Social existence determines social consciousness, and every idea has its specific sociohistorical conditions. With the development and changes of the times, the productivity and mode of production have undergone great changes, and with them some spiritual civilization and idea concepts are not comparable [16]. The emergence and development of Chinese traditional culture are accompanied by the soil that nurtured his growth all the way. As far as it seems now, some traditional cultures are incompatible with the socialist market economy, democratic politics, advanced culture, and social governance [17]. The time has changed, the original cultural connotation should be expanded, improved, and changed, and the excellent Chinese traditional culture should be given a new connotation of the times. In addition, traditional culture is profound and profound, some words are difficult to pronounce, cultural symbols are obscure and difficult to understand, and the word expressions and spiritual concepts are very different from contemporary society, which increases the difficulty of understanding and accepting, making it difficult for traditional culture to be widely spread.

Today's world has entered the information age, and the concept of “Internet+” is deeply rooted in people's minds, and people are committed to finding a faster and more convenient way to achieve their goals [18]. This is especially true for the intake of knowledge, where the problem is solved by “Baidu,” even if the corresponding knowledge is not absorbed, and many times know what is right but not what is wrong, which is not conducive to the understanding and digestion of culture [19]. At the same time, the rise of the Internet and short videos has created a more impatient psychological state of modern people, people are often accustomed to fragmented information. It is difficult to quietly study a book or learn a skill. Many traditional crafts and classical culture are therefore annihilated in the long history, and many excellent traditional culture out of the human stage.

### 2.2. Application of Big Data Technology in the Development of Traditional Culture

In today's era, the influence of big data on people is expanding day by day [20]. In the environment of big data, information and culture are continuously disseminated, copied, drawn, and applied in an accelerated manner, and the cultures of ancient and modern China have never been in such a fierce collision and intersection as today. China's excellent traditional culture is rich in content and is the most brilliant star in the world's cultural treasury. In the process of promoting traditional culture, it is necessary to empower technology, adopt modern information technology and use new media to transmit traditional culture to everyone's life, so that they can comprehend and inherit the excellent traditional culture in a subtle way.

Chinese traditional culture has a long and profound history. The Yuanmingyuan in Beijing, the Terracotta Warriors in Shaanxi, and the Yungang Grottoes in Shanxi have always played an important role in showing the original appearance of monuments and passing on traditional culture. In recent years, with the rapid development of science and technology and the improvement of public cultural needs, artificial intelligence technology represented by VR and AR has been applied to traditional cultural scenic spots, and the continuous transformation of virtual and reality has changed the previous monotonous scenic tour mode and given new vitality to traditional cultural scenic spots. By wearing professional equipment, visitors get a virtual perception of visual, auditory, and tactile aspects [21]. Despite being in the real world, visitors can travel to the virtual world, talk with vivid and realistic historical figures, appreciate the exquisite historical architecture, and feel the livelihood of people in a specific historical environment. This immersive experience can help them get closer to the real traditional culture, enhance interaction and communication, and strengthen their knowledge and understanding of traditional culture [22].

Unlike VR technology, AR is a technology that applies virtual images to the real world. Take Hongkeng Tulou Scenic Area in Yongding District, Longyan City, Fujian Province, for example, it uses AR technology to integrate the real environment and virtual images into the same time and space [23]. Visitors can visually appreciate the whole process of Hongkeng Tulou from the beginning of preparing the foundation to the final ramming of the Earth and building the walls on-site. This technology can not only show the peculiar structure of the ancient building complexes that coalesce the wisdom of ancient working people but also break the time and space limitation, helping visitors to fully understand the history and culture of the Tulou scenic spot [24]. During the tour, visitors can also scan specific signs to obtain 3D exhibit models with sound, text, and special effects so that the cold exhibits are presented in a vivid way in front of visitors, making traditional culture audible and accessible.

“Deep learning technology” refers to the use of artificial intelligence learning ability, automatic recognition of text, image, and sound data, so that the machine learns to learn itself [25]. In recent years, with the development of multiculturalism, some young people think that traditional culture is “not up to date” and have misconceptions about traditional culture, which has led to a narrow audience for traditional culture and affected the development of innovation [26]. Therefore, we need to use the artificial intelligence technology represented by “deep learning technology” to inject contemporary elements into traditional culture, interpret the profound connotation of traditional culture in the form of songs and dances, films, and TV works that young people like, and wrap the profound core of traditional culture with contemporary outer clothing.

Accurate recommendation technology is a new media platform based on the analysis of the audience's likes and dislikes, accurate and personalized dissemination of content of interest to the audience. Chinese traditional culture is all-encompassing and different audiences have different life backgrounds and values. If we lack understanding of audiences' interests and cultural needs and simply promote traditional culture in a “one-size-fits-all” manner, it may cause audiences to resist and get bored, which may have the opposite effect [27]. The precision push technology in the new media app solves this problem. It first collects audience information with the help of big data technology, and then uses artificial intelligence technology to accurately predict the audience's cultural interests, analyze the audience's usage habits, and formulate personalized communication strategies, so as to realize the unification of supply and demand between pushing content and audience needs, and improve the accuracy and effectiveness of traditional culture communication [28]. Take Jiyin App as an example, it combines the use of big data algorithm and artificial autonomous selection technology to push traditional culture related to the elements of interest to the audience in a timely manner and promote the dissemination of traditional culture [29]. For example, if you love dressing, then you will receive videos about traditional clothing; if you are keen on music, then you will receive videos about traditional musical instruments such as the guqin and pipa; if you like food, then you will receive videos related to traditional old-fashioned food. It is Jitterbug's ability to clarify the audience's likes and dislikes that has enabled it to spread traditional Chinese culture and enhance its influence while satisfying the audience's desire to watch [30].

## 3. Algorithm Design

Recommendation system is a kind of information service technology to solve the problem of information overload, which can provide specific information and services that meet the interests and needs of a specific user at a specific time from a large amount of information. With the development of the network, personalized recommendation technology has been widely used in e-commerce, advertising push, and movie recommendation with large amount of information. In order to make traditional cultural resources better disseminated and popularized in the network, it is necessary to use personalized recommendation technology to push traditional cultural news or knowledge to users.

### 3.1. Problems with Traditional Collaborative Filtering Recommendation Techniques

Nowadays, personalized recommendation technology has been put into use on many e-commerce websites. However, with the increase of the number of products and users, the complexity of information is increasing and the bane of the system is decreasing, the personalized recommendation system is facing a great challenge.

As the number of users and items in the system grows substantially, and the process of evaluating items by users is independent of the process of browsing and selection by users, it leads to the fact that users seldom evaluate items, which finally causes the evaluation rate of items to drop so that it results in sparse evaluation data. The similarity neighbor distance obtained by collaborative filtering to calculate similarity in this case is very inaccurate.

The collaborative filtering recommendation technique relies on user rating data of items to generate recommendations. So when a new item is added to the system without rating data, the item cannot be recommended. Similarly, when a new user joins the system without relevant rating information, the system cannot generate accurate recommendations to him/her.

### 3.2. Improvement of Association Rule-Based Recommendation Technology

Association rule mining on the data is performed in two phases, the first phase mines the set of frequent items, and the second phase mines the association rules.

The algorithm flow of Apriori is as follows: first, find the frequent 1-item set, denoted as *L*_1_. Use *L*_1_ to find the frequent 2-item set *L*_2_, while *L*_2_ to find *L*_3_, and so on, until no frequent *k*-item set is found.

The join step first finds *L*_2_ and produces the set *C*_*k*_ of candidate *k*-item sets by self-join. Perform *L*_*k*−1_ join, where the elements of *L*_*k*−1_ are joinable if their first *k* − 2 items are the same, that is, (*l*_1_[1]=*l*_2_[1])∧(*l*_1_[2]=*l*_2_[2])∧…∧(*l*_1_[*k* − 2]=*l*_2_[*k* − 2])∧(*l*_1_[*k* − 1] < *l*_2_[*k* − 1]). The condition *l*_1_[*k* − 1] < *l*_2_[*k* − 1] is a guarantee that no duplicates are generated. Joining *L*_1_ and *L*_2_ produces the resultant set of terms *l*_1_[1]*l*_2_[1] … *l*_1_[*k* − 1]*l*_2_[*k* − 1]. The algorithm complexity is

(1) $$\begin{array}{c} C=L_{1}^{\prime }*L_{1}^{\prime }+L_{2}^{\prime }*L_{2}^{\prime }+L_{k-1}^{\prime }*L_{k-1}^{\prime }+L_{k}^{\prime }*L_{k}^{\prime }, \end{array}$$

where *L*_*k*_′ is the number of frequent terms containing *k* terms.

Due to

(2) $$\begin{array}{c} L_{1}^{\prime }\leq C_{n}^{1},L_{2}^{\prime }\leq C_{n}^{2},\ldots ,L_{k}^{\prime }\leq C_{n}^{k}\left(k\leq n\right). \end{array}$$

So the result of (1) is at most:

(3) $$\begin{array}{c} C_{n}^{1}*C_{n}^{1}+C_{n}^{2}*C_{n}^{2}+C_{n}^{n-1}*C_{n}^{n-1}+C_{n}^{n}*C_{n}^{n}=C_{2n}^{n}-1. \end{array}$$

Therefore, the algorithmic complexity of the join step is O(*C*_2*n*_^*n*^).

It is easy to see that the complexity of the algorithm of the join step is the complexity of the whole Apriori algorithm. An algorithm of this order of complexity will run extremely low in application. To address this problem, an improved algorithm is proposed by conducting extensive research and verification on the join step.

### 3.3. Improve the Architecture and Flow of the Algorithm

**Figure 1:**
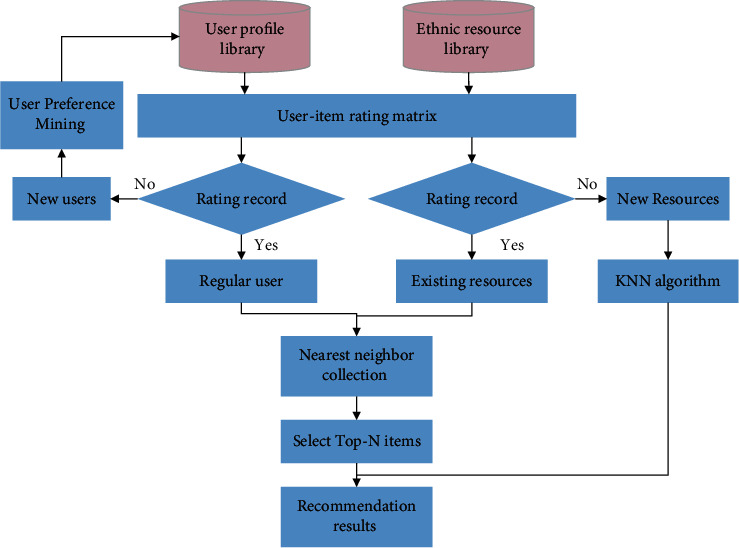
Flow chart of improved collaborative filtering traditional cultural resources hybrid recommendation algorithm.

**Figure 2:**
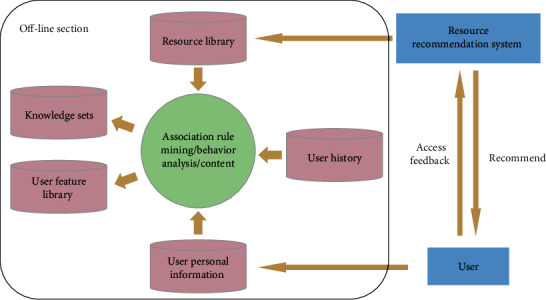
Architecture of the offline part.

The improved collaborative filtering traditional cultural resource recommendation algorithm uses a hybrid recommendation based on collaborative filtering, content filtering, and association rules to alleviate the data sparsity and cold-start problems. The collaborative filtering recommendation system is based on the results of the user's rating matrix for items to be recommended and uses a predictive rating filler matrix to improve the problem of inaccurate recommendation results caused by the sparse matrix. The content filtering recommendation system does not have the cold-start problem, which is combined with the content filtering based on KNN algorithm when new items enter the system to compensate for the cold-start problem of new items. Establish a user feature database, collect user preferences when users register, and mine the association rules for user preferences. Users with high similarity to new user preferences are found in the user features to improve the new user cold-start problem. The main process of traditional cultural resource recommendation algorithm is shown in Figure 1:

Figure 2 shows the offline part of the improved collaborative filtering minority resource recommendation algorithm. The computational process of the offline part of the improved algorithm is mining the user's personal information using association rules to derive the user's feature base, which provides a solution rule for the cold-start problem of new users. Behavior analysis is performed on user behavior records to implicitly rate users and solve the data sparsity problem; content filtering is performed on the content in the ethnic resource library to compensate for the item cold-start problem in the collaborative filtering algorithm. Separate a large number of calculation processes in the offline part, the user only needs to access and feedback to the system when online, and the system can quickly recommend the results to the user through the data completed by the calculation when offline, which greatly reduces the waiting time of the user for recommendation and improves the real-time of the system.

**Figure 3:**
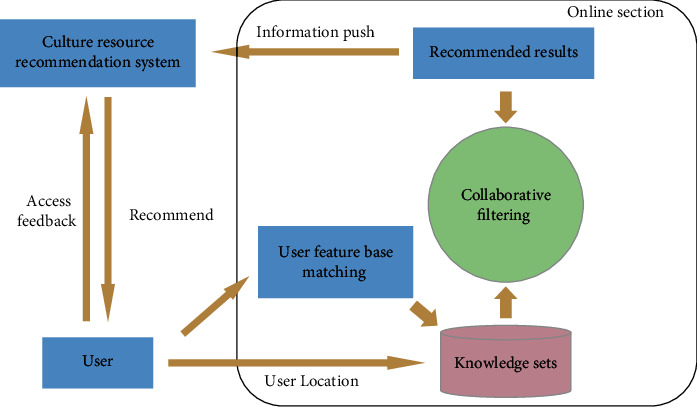
Online section architecture.

After moving the complex calculations to the offline part, the online part is simpler. By analyzing the complete knowledge set and using the collaborative filtering algorithm, the improved collaborative filtering recommendation results can be obtained quickly. By using the association rule-based recommendation algorithm, the association rules in the knowledge set are matched and the association rule-based recommendation results are obtained. The final results of the traditional culture resource recommendation algorithm are obtained by combining and ranking the results of the two algorithms. The architecture of the online part of the traditional cultural resource recommendation algorithm is shown in Figure 3. After the online part is based only on the user's demand, the system uses the results in the knowledge set calculated by the offline part through the user's demand and uses the improved collaborative filtering algorithm to derive the recommendation results. Finally, the information in the result is pushed to the user, completing the whole process of the traditional culture resource recommendation system.

During the actual use of the system, it is inevitable that users will rate the resource items after browsing the recommended information. This behavior is also a reason for the sparse scoring matrix. In order to remedy this problem, there are two ways to obtain users' ratings of resource items: one is an explicit rating mechanism to obtain direct ratings through users' ratings of browsing resource items; the other is to obtain users' preferences for items implicitly through their behavior of browsing resource items, which is converted into ratings.

## 4. Experiments

### 4.1. Experiment Preparation

Mean absolute error (MAE) is the deviation between predicted and actual ratings calculated by absolute value, and the smaller the deviation value, the more accurate the prediction. Suppose the set of user ratings for items is {*P*_1_, *P*_2_,…, *P*_*n*_}, and the set of predicted user ratings for unrated items is {*Q*_1_, *Q*_2_,…, *Q*_*n*_}, then MAE is expressed as (4). In addition, common accuracy and recall rates are used as evaluation metrics in this paper:

(4) $$\begin{array}{c} \text{MAE}=\frac{\sum_{i=1}^{n}\left|Q_{i}-P_{i}\right|}{n}. \end{array}$$

The data used in this paper are from the internal nonpublic data of the Chinese Traditional Culture Creation and Development Research Center. The experiment selected 3666 users as the target users to be tested, and 80% of the dataset item scores were used as the training dataset, while the remaining 20% of the data were used as the test set for the final results, and finally the comparison between the actual and predicted values was performed. The data set details are shown in Table 1. The experiments in this paper were conducted on a Dell T7920 workstation with an NVIDIA RTX1080TI graphics card and 32G RAM. The Adam optimizer is used, the batch parameters are set to 16, the number of training rounds is 20, and the learning rate is 0.0001 in the first 10 training rounds and decays linearly from 0.0001 to 0 in the second 40 training rounds.

**Figure 4:**
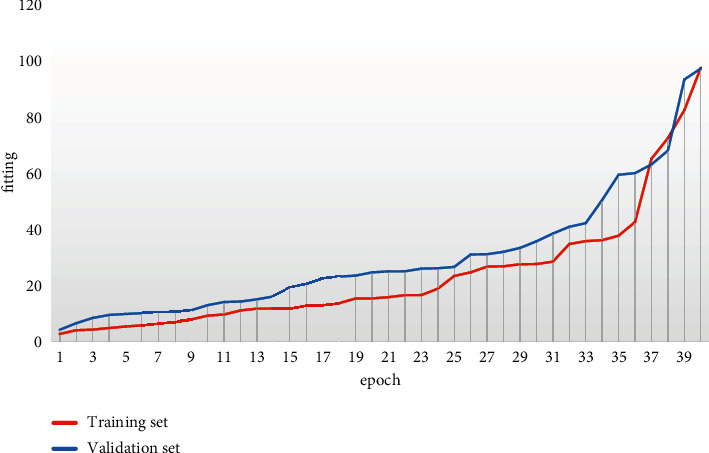
Schematic diagram of training process performance improvement.

**Figure 5:**
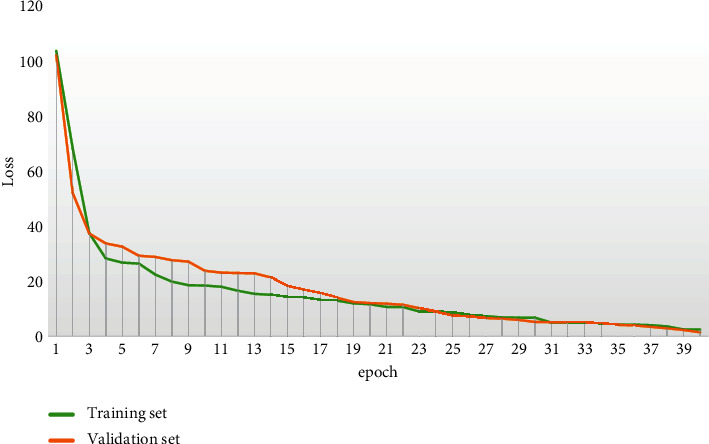
The training process loss convergence schematic.

The training process performance enhancement and loss convergence are shown in Figures 4 and 5.

### 4.2. Performance Analysis of Improved Collaborative Filtering Techniques

To test the correctness and efficiency improvement of the algorithm, 60 of the data were selected for the experiments. The correctness of the improved algorithm was demonstrated by experimenting with the same running results of the traditional collaborative filtering technique and the improved algorithm program based on association rules.  Table 2 shows the time comparison generated when the support threshold is set to 30. From the data in the table, it can be seen that the improved algorithm has improved in efficiency, thus also proving the feasibility of the improved algorithm.

The improved collaborative filtering resource recommendation algorithm and the traditional collaborative filtering recommendation algorithm were tested experimentally according to the above MAE method to visualize the accuracy of recommendations. A total of five rounds of experiments were conducted, and the experimental results are shown in Table 3. It can be concluded that the improved collaborative filtering algorithm has higher recommendation quality.

**Figure 6:**
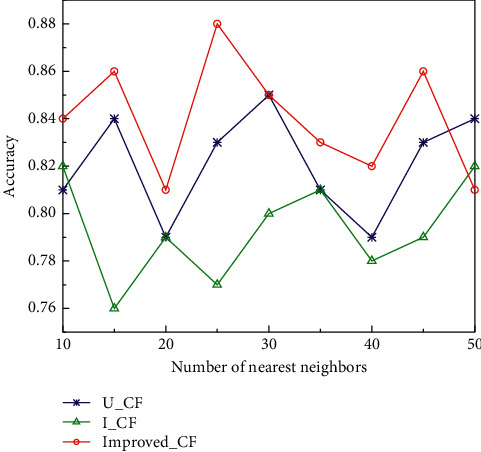
Effect of different number of nearest neighbors on accuracy.

**Figure 7:**
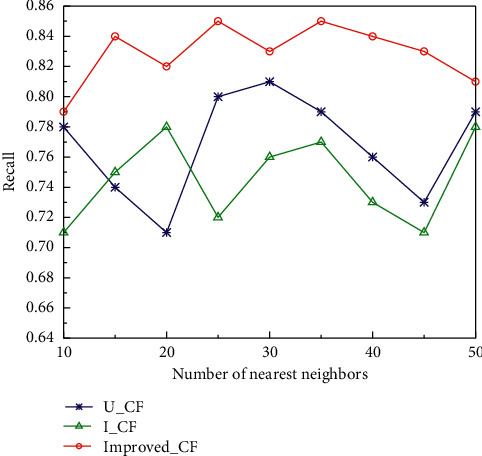
Effect of different number of nearest neighbors on recall rate.

In the experiments, the algorithms take the number of nearest neighbor user sets as 50, 45, 40, 35, 30, 25, 20, 15, 10, respectively, and the average values of accuracy and recall are obtained ten times for the experiments, and the effects of accuracy and recall of the three algorithms are shown in Figures 6 and 7. U_CF denotes the user-based collaborative filtering algorithm; I_CF denotes the item-based collaborative filtering algorithm; Improved_CF denotes the improved algorithm. It can be clearly seen that the accuracy and recall of all three algorithms change significantly with the expansion of the number of nearest neighbors. Improved_CF algorithm shows an increasing trend of recall and accuracy at the beginning with the increase of the number of nearest neighbors. With the change of the number of nearest neighbors, the recall and accuracy show a phase decrease and rebound. When the number of nearest neighbors is small, the recall of the Improved_CF algorithm is small, but with the gradual increase of the number of nearest neighbors, the recall starts to rebound.

The comparison between the improved algorithm and the traditional algorithm is shown in Table 4, which shows that the improved algorithm has 8.56% and 5.09% higher accuracy and recall, respectively, compared with the user-based collaborative filtering algorithm, and 9.74% and 8.91% higher accuracy and recall, respectively, compared with the item-based collaborative filtering algorithm.

**Figure 8:**
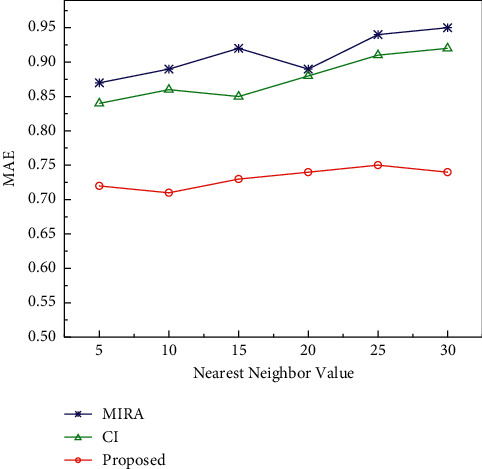
Comparison of MAE of different algorithms.

In addition, we compared the mean absolute error of the algorithm designed in this paper with the traditional collaborative filtering multinode information resource allocation recommendation algorithm (MIRA) and the collaborative filtering recommendation algorithm with contextual information (CI). The results are shown in Figure 8. The average absolute errors of the three algorithms do not show a pattern of getting larger with the increase of the proximity value. However, the average absolute error of the algorithm designed in this paper is between 0.70 and 0.75, which is the smallest error among the three algorithms.

## 5. Conclusions

As an ancient civilization with a history of 5,000 years, China's traditional excellent culture is integrated into the national bloodline, is the spiritual driving force of the Chinese nation to survive, of which the cultural heritage is deservedly the world's first. The era of big data is an unstoppable historical trend, and traditional culture should play its respective role. Using advanced science and technology to dig deeper into the new era value of traditional culture, big data should continue to help the heritage of traditional culture innovation, but should not be overly as traditional culture should insist on improving its own level of cultural value and cultural vitality, making good use of the current artificial intelligence, cloud computing, new media, and other means to carry out deep expansion and absorb the new momentum of the times, so as to achieve its own harmonious development and long-term sustainable development, and let the cultural treasures of the Chinese nation be passed on forever. In this paper, we propose a hybrid recommendation technology based on association rules to improve the traditional collaborative filtering technology. The experimental results show that the recommendation accuracy is higher and the robustness is stronger compared with the traditional method before improvement. In the future, we plan to use recurrent neural networks to explore the path of innovative development of traditional culture under big data.

## Figures and Tables

**Table 1:** Distribution of experimental data.

ID	Training set	Test set
Number of users	2933	733
Number of projects	188	47
Number of ratings	18902	4726
Sparsity rating	0.963	

**Table 2:** Comparison of the time to generate frequent *k*-item sets between the traditional algorithm and the improved algorithm

Method	Time consumption (s)			
	Frequent itemset 1	Frequent itemset 2	Frequent itemset 3	Frequent itemset 4
Traditional algorithm	966	5087	100097	683199
Improved algorithm	**583**	**3294**	**6712**	**236173**

**Table 3:** Comparison of MAE between traditional recommendation technique and improved traditional cultural resource recommendation technique.

Method	Number of assessment groups				
	1	2	3	4	5
Traditional algorithm	1.04	1.09	1.01	1.10	1.01
Improved algorithm	**0.86**	**0.82**	**0.84**	**0.88**	**0.85**

**Table 4:** Comparison of the results between the improved algorithm and the traditional algorithm.

	Accuracy (%)	Recall (%)
U_CF	80.56	79.28
I_CF	79.38	75.46
Improved_CF	**89.12**	**84.37**

## Data Availability

The datasets used during the current study are available from the author upon reasonable request.
